# Silencing LncRNA HCG27 ameliorates cognitive dysfunction after ischemic stroke via miR-27a-3p regulation

**DOI:** 10.1186/s41065-025-00493-6

**Published:** 2025-07-18

**Authors:** Ting Li, Ying Li, Lin Chen, Chaosheng Zeng, Huaijie Xing, Min Chen, Limin Yan, Xiaopei Zhang

**Affiliations:** 1 Department of Neurology, The First People’s Hospital of Shenyang, Shenyang, 110041 China; 2 The Tenth Sanatorium Department, Qingdao Special Service Sanatorium of PLA Navy, Qingdao, 266071 China; 3 Department of Neurology, The Second Affiliated Hospital of Hainan Medical University, No. 368, Yuhai Avenue, Longhua District, Haikou, 570311 Hainan Province China; 4 Hainan Medical University, Haikou, 571199 Hainan Province China; 5 Department of Neurology, Affiliated Hospital of Nantong University, No. 20, Xisi Road, Chongchuan District, Nantong, 226001 Jiangsu Province China

**Keywords:** HCG27, miR-27a-3p, Cognitive dysfunction, Cerebrovascular disease, Cerebral ischemia-reperfusion

## Abstract

**Background:**

We explore the effect of improving cognitive dysfunction after cerebral ischemia–reperfusion (CI/R) by regulating HCG27.

**Methods:**

The MCAO and OGD/R methods were employed to establish *in vivo and in vitro* models of cognitive dysfunction caused by a CI/R injury. RT-qPCR was utilized to detect the relative expression of HCG27 and miR-27a-3p. An ELISA was adopted to measure the concentrations of inflammatory factors (IL-6, IL-1β, IL-10). The concentration of MDA and activity of CAT were detected using commercially available kits. The neurological deficit was evaluated using the mNSS score. The spatial learning and memory capabilities were evaluated via the MWM test. The targeting relationships were validated by the dual-luciferase reporter assay, RIP assay, and RNA pull-down assay. The CCK-8 assay and flow cytometry were employed to asses cell viability and apoptosis, respectively.

**Results:**

The level of HCG27 was upregulated in MCAO rats and OGD/R-induced BV2 cells, whereas that of miR-27a-3p decreased, and HCG27 targeted miR-27a-3p. Compared with the sham group, the mNSS score of MCAO rats was elevated, and their spatial learning and memory abilities declined, with aggravated inflammatory response and oxidative stress. However, silencing HCG27 improved these conditions, and the miR-27a-3p antagonist reversed this. MiR-27a-3p reversed the increase in cell viability in OGD/R-induced BV2 cells, reduced the cell apoptosis rate, and weakened the inflammatory response and oxidative stress caused by the HCG27 silencing.

**Conclusions:**

Silencing HCG27 can protect against cognitive dysfunction after cerebrovascular disease by targeting miR-27a-3p.

**Supplementary Information:**

The online version contains supplementary material available at 10.1186/s41065-025-00493-6.

## Background

Ischemic stroke is a type of acute cerebrovascular disease with a high mortality rate and morbidity. It is one of the primary diseases endangering human health [1]. Currently, cerebral ischemia–reperfusion (CI/R) is an effective treatment for ischemic stroke [2]; however, it can also cause dysfunction and structural damage to tissues and organs, resulting in a CI/R injury [3]. Cognitive dysfunction is one of the complications of CI/R injury after ischemic stroke, which affects the lives of patients [4]. Hence, it is indispensable to explore the regulatory mechanism of cognitive dysfunction caused by CI/R injury.

Long non-coding RNAs (lncRNA), a class of regulators involved in biological functions in the form of RNAs [5], have been extensively investigated recently for their involvement in neurodegenerative diseases [6]. Downregulation of NLRP3 by silencing lncRNA 4344, targeting miR-138-5p, to modulate associated neuroinflammation and cognitive deficits [7]. The silencing of lncRNA SIX3OS1 facilitated neurological recovery in patients with post-stroke cognitive impairment by targeting the miR-511-3p/RBP4 axis [8]. The PI3K/Akt signaling pathway can be inhibited by up-regulation of lncRNA MEG3, which mitigated the cognitive deficits and pathological damage to hippocampal neurons apoptosis in rats with Alzheimer’s disease (AD) [9].Transcriptome profiling revealed that lncRNA HCG27 differentially expressed in ischemic stroke [10]. In addition, competitive endogenous RNA network analysis revealed that HCG27 was differentially expressed in acute ischemic strokes and may correlate with inflammation [11]. HCG27 might be related to inflammation in subarachnoid hemorrhage caused by a ruptured intracranial aneurysm [12]. While lncRNAs like NLRP3 and SIX3OS1 are studied in CI/R, the role of HCG27 in cognitive dysfunction remains entirely uncharacterized.

In this study, we established a rat model of cognitive dysfunction generated by CI/R injury by middle cerebral artery occlusion (MCAO). In addition, an in vitro cell model was constructed by inducing BV2 cells with oxygen-glucose deprivation/reoxygenation (OGD/R). We primarily investigated the function of HCG27 in the cognitive regulation of MCAO rats and its influence on the injury of BV2 cells. We explored the role of HCG27 in the cognitive regulation of MCAO rats and its influence on BV2 cell injury. This study is the first to link HCG27 to miR-27a-3p in regulating neuroinflammation and oxidative stress after CI/R.

## Methods

### Establishment of a cognitive dysfunction model

All animal experimental protocols were approved, and all experiments were performed according to the guidelines of the Animal Ethics Committee of the Second Affiliated Hospital of Hainan Medical University (LW20210324). The study was conducted in agreement with the ARRIVE guidelines. Male Sprague–Dawley rats (age: 7–8 weeks, weight: 200–240 g) were purchased from the Animal Center of Dalian Medical University. The rats were housed at 20–23℃ with a relative humidity of 55% in the housing. MCAO was used to establish a rat model of cognitive dysfunction. The rats were anesthetized and disinfected, exposing the left common carotid artery of the rat and making a small incision. A nylon filament was inserted through an incision to occlude the left middle cerebral artery. Specifically, a 4 − 0 nylon filament with a rounded tip (diameter: 0.26 mm) was inserted into the internal carotid artery to a depth of ~ 18–20 mm from the common carotid artery bifurcation to occlude the middle cerebral artery origin. The nylon filament was removed 1 h later, and the incision was sutured. Furthermore, surgical procedures other than the obstruction treatment were applied to the rats in the sham group.

### Grouping

Eighty rats were randomly divided into two groups. One group consisted of 32 rats, which were randomly divided into 4 subgroups for was investigating the function of lncRNA HCG27 in rat nerve injury, with specific sub-grouping as follows: sham, MCAO, MCAO + sh-NC, and MCAO + sh-HCG27. The second group consisted of 48 rats, was used to analyze the synergistic effects of HCG27/miR-27a-3p in cognitive dysfunction in rats, and further divided into the following sub-groups: sham, MCAO, MACO + sh-NC, MCAO + sh-HCG27, MCAO + sh-HCG27 + antagomir NC, and MCAO + sh-HCG27 + miR-27a-3p antagomir. Rats were randomly assigned to experimental groups using a computer-generated random number table. The investigator responsible for surgery and behavioral testing was blinded to the grouping until all data were collected and analyzed. Conducted a power analysis using G*Power software (v.3.1.9.7) based on pilot data (effect size d = 2.21, α = 0.05, 1-β = 0.8), confirming that *n* = 8 animals per group provides appropriate power to detect significant differences in behavioral and biochemical endpoints.

### Modified neurologic severity scores (mNSS)

The mNSS was used to evaluate the degree of neurological function impairment. The evaluators were unaware of the grouping situations of each group. The evaluations were conducted on the 0th day, 1st day, 3rd day, and 7th day after the establishment of the cognitive dysfunction model. The evaluation primarily focused on motor function, sensory function, reflex response tests, and balance tests. The scoring range was from 0 to 18 points. Among them, the higher scores indicated more severe neurological impairment.

### Morris water maze (MWM) test

The experiment assessed learning for location navigation and memory for spatial exploration. Opaque water was added to a circular tank (180 cm diameter, 80 cm height) at 23–25℃. A platform was placed in one of the four quadrants of the pool, which was split into four quadrants. We conducted the localization navigation experiment in which the rats were acclimatized to the pool environment on day 0. On days 1–5 of the experiment, the rats were placed into a certain quadrant in a quasi-random manner. The time taken by the rats to identify the hidden platform, also known as the latency period, was observed and recorded. In addition, we conducted the space exploration experiment on day 6, in which the platform was removed, and the rats were allowed to swim freely for 90 s, and the time for which the rats stayed in the target quadrant and the number of times they crossed the platform were recorded. The behavior of the rats was tracked and analyzed using the ANY-maze software. Assessors were blinded to the grouping conditions, and the data were analyzed by independent observers. The rats were anesthetized with 3% sodium pentobarbital after the test was completed, and the serum was collected. The rats were anesthetized, and euthanized by cervical dislocation. Finally, the brain tissues were removed and stored at − 80℃.

### Cell culture and treatment

BV2, murine microglial cells were cultured in Dulbecco’s Modified Eagle’s Medium (DMEM). Glucose-free DMEM was used as the cell culture medium when the cells were in the logarithmic growth phase. Next, the cells were cultured under anaerobic conditions with 5% carbon dioxide and 95% nitrogen for 4 h, respectively, and subsequently transferred to aerobic conditions and cultured in a normal culture medium for 0, 6, 12, and 24 h [13].

### Real-time quantitative PCR (RT-qPCR)

Total RNA in the cells and serum was extracted using the TRIzol reagent. PrimeScript™ RT kit and TaqMan miRNA Reverse Transcription kit were utilized to reverse transcribe lncRNA HCG27 and miR-27a-3p into cDNA, respectively. Next, PCR amplification was performed using the Takara SYBR Super Mix Kit. GAPDH and U6 served as internal references for lncRNA HCG27 and miR-27a-3p respectively. Set three biological replicates for each group. Primer sequences for HCG27, miR-27a-3p, GAPDH, and U6 are listed in Supplementary Table S1. RT-PCR reactions were performed under the following conditions: 94℃, 2 min; 35–40 cycles of 94℃ for 30 s, gene-specific annealing temperature for 30 s, and 72℃ for 60 s. Finally, the 2⁻^ΔΔCt^ method was adopted for data normalization.

### Cell viability and apoptosis detection

The treated BV2 cells were suspended and seeded into the plate, and subsequently cultured under normal conditions (37 °C, 5% CO₂) for 0, 24, 48, and 76 h, respectively. with the cells were incubated in the CCK-8 solution, and the absorbance at a wavelength of 450 nm was determined using a microplate reader.

The BV2 cells were digested with trypsin, washed, centrifuged and resuspended. Next, 100 µL of the cell suspension was taken to which 5 µL of Annexin V–fluorescein isothiocyanate and 5 µL of propidium iodide were added, and the mixture was incubated in the dark. Finally, the cell apoptosis rate was evaluated by flow cytometry.

### Enzyme-linked immunosorbent assay (ELISA)

The concentrations of interleukin (IL)-6, IL-1β, and IL-10 were detected using ELISA kits. Plasma and cell supernatants were added to the microplates and incubated with anti-biotin antibodies for 2 h. Finally, the chromogenic solution was added, and the absorbance value was calculated using a microplate reader.

### MDA and CAT detection

BV2 cells and brain tissues were added to a precooled phosphate buffer, homogenized, and centrifuged to collect supernatants. Malondialdehyde (MDA) level and catalase (CAT) activity were assessed following the instructions provided in the MDA and CAT kits.

### Bioinformatics analysis

The lncLocator database was used to analyze the location of HCG27 in cells. The DIANA and LncBook databases were used to prognosticate the target microRNAs (miRNAs) of HCG27. The binding sites were analyzed using LncBook 2.0.

### Dual-Luciferase reporter assay

Wild-type plasmid HCG27 WT and mutant plasmid HCG27 MUT were constructed. The BV2 cells were seeded into a 6-well plate. Lipofectamine 2000 and the aforementioned plasmids, together with miR-27a-3p mimic and mimic negative control (mimic NC), were co-transfected into the cells. The luciferase activity was detected after 48 h of culture.

### RNA Immunoprecipitation (RIP) assay and RNA pull-down assay

BV2 cells were lysed, followed by an 8-h incubation with magnetic beads conjugated to either anti-Ago2 antibody or IgG antibody. The enrichment levels of HGC and miR-27a-3p were subsequently determined using RT-qPCR.

The biotin-labeled negative control and miR-27a-3p mimic (denoted as bio-NC and bio-miR-27a-3p respectively) were incubated with streptavidin beads. Next, the cells were lysed, and the RNA complexes pulled down by the beads were collected. Finally, RT-qPCR was used to detect the content of HCG27 in the RNA complexes.

### Statistical methods

The data were statistically analyzed were conducted using SPSS 23.0 and GraphPad Prism 9.0. Data were tested for normality using the Shapiro-Wilk test. For normally distributed data, one-way ANOVA with Tukey’s post-hoc test was used for multiple group comparisons, and Student’s t-test for two-group comparisons. Significance was established when the P-value was < 0.05.

## Results

### HCG27 Silencing alleviated nerve injury in MCAO rats

**Fig. 1:**
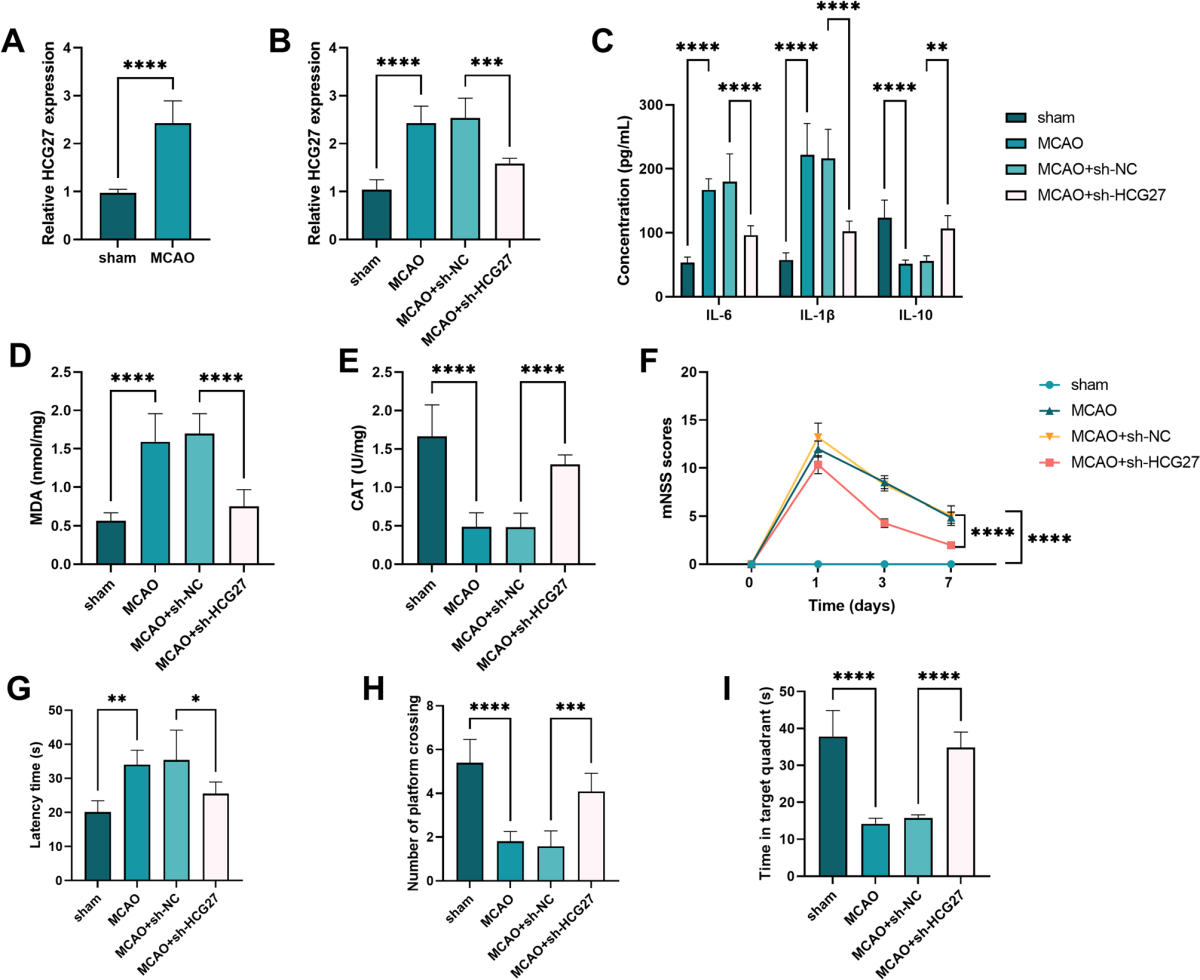
Silencing of lncRNA HCG27 improves cognitive dysfunction and inflammatory injury in MCAO rats. **A-B.** The relative expression of HCG27 in MCAO rats. **C.** The concentration of IL-6, IL-1β, and IL-10 in MCAO rats. **D-E.** The concentration of MDA and the activity of CAT in MCAO rats. **D.** The mNSS score of rats. **G-I.** The latency time, number of platforms crossed, and time in the target quadrant of rats in the MWM experiment, *n* = 8 rats per group. *P < 0.05, **P < 0.01, ***P < 0.001, and ****P < 0.0001

The level of HCG27 in MCAO rats was notably upregulated compared with that in the sham group (*P* < 0.0001, Fig. 1A). However, this upregulation was inhibited by sh-HCG27 (*P* < 0.001, Fig. 1B). ELISA experiment revealed that the concentrations of IL-6 and IL-1β were dramatically downregulated in MCAO rats transfected with sh-HCG27, whereas the concentration of IL-10 was upregulated (*P* < 0.01, Fig. 1C). Moreover, MCAO rats had increased MDA levels and decreased CAT levels compared with the sham group, both of which were reversed by Sh-HCG27 (*P* < 0.0001, Fig. 1D-E). In addition, the mNSS score of MCAO rats significantly increased, whereas that of MCAO + sh-HCG27 rats decreased (*P* < 0.0001, Fig. 1F). Compared with the sham group, the MCAO rats exhibited a significantly prolonged latency, along with a marked reduction in both the number of platform crossings and the time spent within the target quadrant in MWM experiment. However, the above three indicators were reversed the MCAO + sh-HCG27 rats (*P* < 0.05, Fig. 1G-I).

### HCG27 targeted miR-27a-3p

**Fig. 2:**
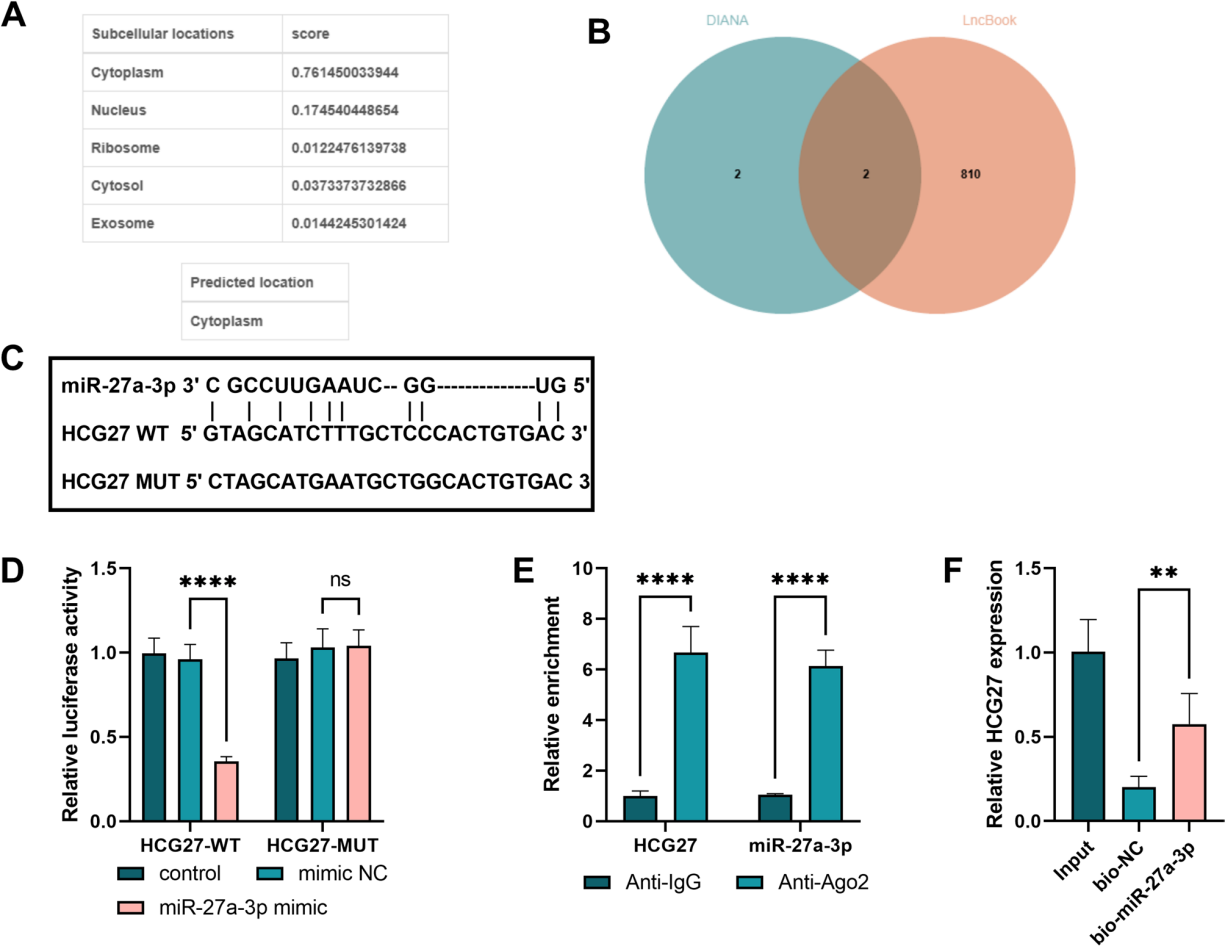
HCG27 target miR-27a-3p. **(A)** The LncLocator database was used to analyze the location of HCG27 in cells. **(B)** Venn diagrams generated by the DIANA and LncBook databases. **(C)** The binding sites between HCG27 and miR-27a-3p. **D-F.** The relationship between HCG27 and miR-27a-3p was detected by the dual-luciferase reporter, RIP, and RNA pull-down assays. **P < 0.01, ****P < 0.0001

To understand the specific mechanism by which HCG27 regulates CI/R injury-induced cognitive dysfunction, we analyzed the subcellular localization of HCG27. HCG27 was predominantly localized to the cytoplasm (Fig. 2A). The DIANA and LncBook databases identified miR-27a-3p as a target microRNA of HCG27 (Fig. 2B). In addition, LnBook 2.0 was used to predict the binding sites between HCG27 and miR-27a-3p (Fig. 2C). The dual-luciferase reporter assay unequivocally demonstrated that the miR-27a-3p mimic significantly attenuated the luciferase activity of HCG27-WT (*P* < 0.0001, Fig. 2D). Moreover, both HCG27 and miR-27a-3p were enriched on the anti-Ago2 magnetic beads with RIP assay (*P* < 0.0001, Fig. 2E). The relative expression of HCG27 in the bio-miR-27a-3p group was elevated compared to that in the bio-NC group in the RNA pull-down assay (*P* < 0.01, Fig. 2F).

### Involvement of the HCG27/miR-27a-3p axis in inflammation and oxidative stress in MCAO rats

**Fig. 3:**
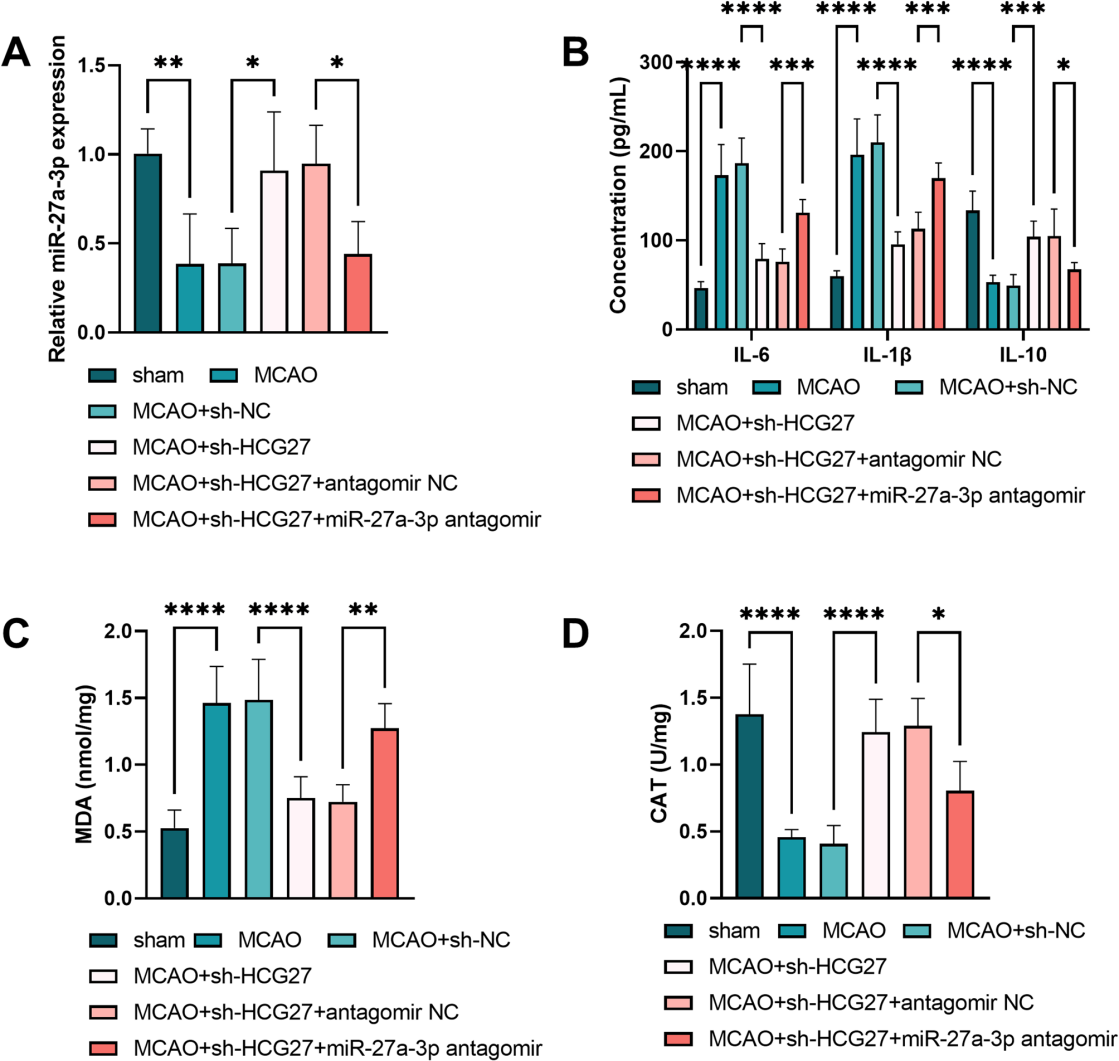
HCG27 participates in the inflammatory response and oxidative stress of rats by targeting miR-27a-3p. **(A)** The level of miR-27a-3p in MCAO rats. **(B)** The concentration of IL-6, IL-1β, and IL-10 in rat serum. **C-D.** The concentration of MDA and the activity of CAT in rats. Data are presented as mean ± SD from *n* = 8 rats per group. *P < 0.05, **P < 0.01, ***P < 0.001, and ****P < 0.0001

We demonstrated that HCG27 CI/R injury upregulated the relative expression of miR-27a-3p, which was effectively reversed by miR-27a-3p antagonist. (*P* < 0.05, Fig. 3A). Furthermore, significant changes occurred in the levels of inflammatory factors and oxidative stress markers in MCAO rats. We have previously reported that silencing HCG27 decreased the concentrations of IL-6 and IL-1β and increased that of IL-10. However, the miR-27a-3p antagonist promoted the concentration of IL-6 and IL-1β and inhibited IL-10 (*P* < 0.05, Fig. 3B). Similarly, the level of MDA and activity of CAT were reversed by the miR-27a-3p antagonist in MCAO rats with silenced HCG27 (*P* < 0.05, Fig. 3C-D).

### Role of the HCG27/miR-27a-3p axis in cognitive dysfunction of MCAO rats

**Fig. 4:**
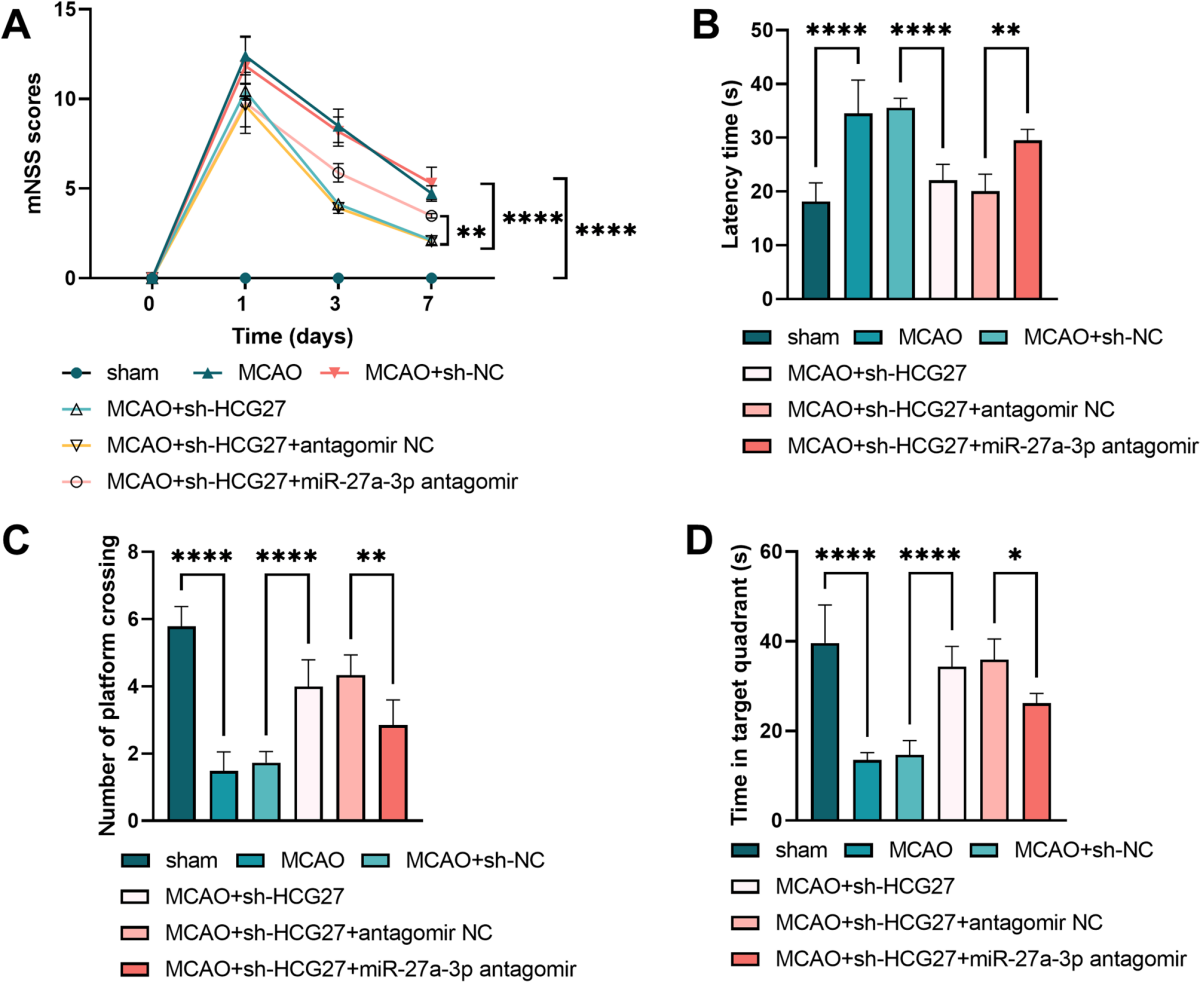
HCG27 acts on cognitive dysfunction in rats by targeting miR-27a-3p. **A.** The degree of nerve injury was assessed using the mNSS score. **B-D.** The latency time, number of platforms crossed, and time in the target quadrant of rats in the MWM experiment, *n* = 8 rats per group. *P < 0.05, **P < 0.01, and ****P < 0.0001

We next wanted to understand the correlation of the HCG27/miR-27a-3p axis in CI/R injury-induced cognitive dysfunction. The miR-27a-3p antagonist could reverse the decrease in the mNSS score of MCAO rats with silenced HCG27 (*P* < 0.01, Fig. 4A). Similarly, the Morris water maze experiment demonstrated that the phenomena of shortened latency time, increased number of platform crossings, and the time in the target quadrant in MCAO rats caused by silencing HCG27 were notably reversed after reducing the level of miR-27a-3p (*P* < 0.05, Fig. 4B-D).

### Role of the HCG27/miR-27a-3p axis in in vitro nerve injury

**Fig. 5:**
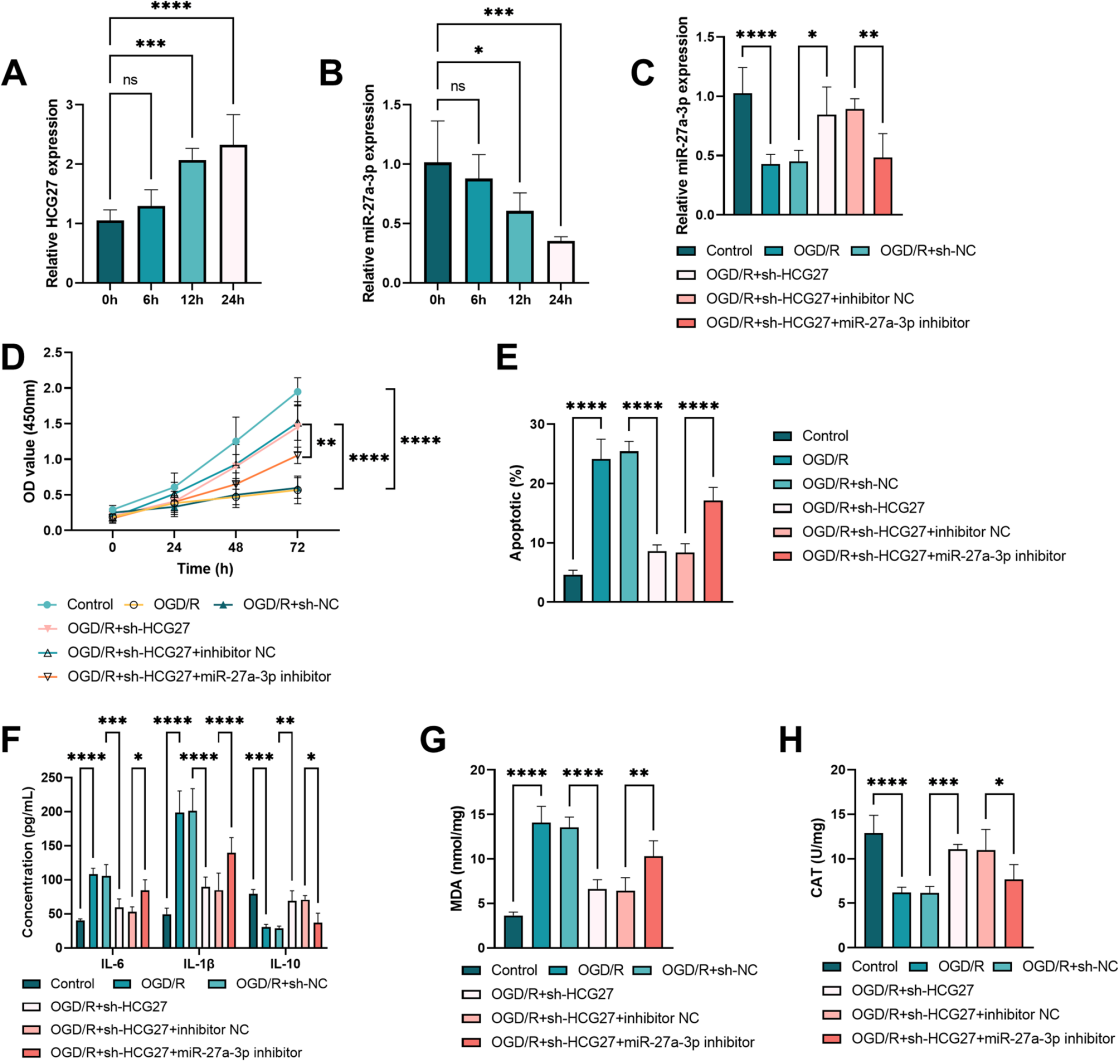
HCG27 participates in the viability, apoptosis, inflammatory response, and oxidative stress in BV2 cells induced by OGD/R by targeting miR-27a-3p. **A-B.** The relative expression of HCG27 and miR-27a-3p in BV2 cells under different reoxygenation times. **C.** The expression of miR-27a-3p in OGD/R-induced BV2 cells. **D-E.** Cell viability and apoptosis. **F.** The inflammatory factors in BV2 cells induced by OGD/R. **G-H.** The concentration of MDA and activity of CAT in OGD/R-induced BV2 cells. Data are presented as mean ± SD from *n* = 3 independent experiments, with each experiment performed in triplicate. *P < 0.05, **P < 0.01, ***P < 0.001, and ****P < 0.0001

We established an in vitro cell model to probe the involvement of the HCG27/miR-27a-3p axis in cell injury caused by OGD/R. The reoxygenation time was prolonged, and the relative expression of HCG27 exhibited an upward trend concomitant with the elongation of reoxygenation duration (*P* < 0.001, Fig. 5A), whereas the expression of miR-27a-3p decreased significantly (*P* < 0.05, Fig. 5B). The miR-27a-3p inhibitor counteracted the relative reduction in the expression of miR-27a-3p resulting from silencing HCG27 in BV2 cells subjected to OGD/R conditions (*P* < 0.05, Fig. 5C). Silencing HCG27 increased cell viability and reduced cell apoptosis. Conversely, the downregulation of miR-27a-3p reversed these effects (*P* < 0.01, Fig. 5D-E). Moreover, sh-HCG27 reduced the concentrations of IL-6 and IL-1β in terms of inflammatory factors and oxidative stress markers, whereas increasing the concentration of IL-10 (*P* < 0.05, Fig. 5F). Nevertheless, the miR-27a-3p inhibitor reversed these effects. Compared with BV2 cells of OGD/R + sh-NC, MDA level was downregulated, and the activity of CAT was upregulated in BV2 cells of OGD/R + sh-HCG27, and these two were reversed by miR-27a-3p (*P* < 0.05, Fig. 5G-H).

## Discussion

The prevalence of cerebrovascular disease has witnessed a continuous increase as the living standards have steadily improved and the population has aged at an accelerating pace [14, 15]. Brain injury caused by CI/R injury can cause cognitive dysfunction in patients [16]. As the most common type of impairment among patients with cerebrovascular diseases, cognitive dysfunction seriously affects the quality of life of patients [17]. Previous studies have implicated lncRNAs in central nervous system diseases. For example, the overexpression of Rain is known to alleviate neuronal damage and apoptosis, and regulate the miR-143-3p/LIMK1 axis, thus playing a neuroprotective role [18]. OIP5-AS1 exerted a neuroprotective effect in aged rats with postoperative cognitive dysfunction (POCD) by targeting miR-186-5p [19]. In addition, lncRNA HCG27 is differentially expressed in ischemic stroke [10, 11]. Therefore, we speculated that the involvement of HCG27 in the development of cognitive dysfunction after cerebral disease. Thus, we constructed an MCAO rat model and conducted a vitro experiment on OGD/R-induced BV2 cells to investigate the role of HCG27 in CI/R injury-induced cognitive dysfunction.

This study demonstrated that HCG27 was notably upregulated in MCAO rats, and its expression in BV2 cells continuously increased as the reoxygenation time prolonged. Inflammatory response and oxidative stress are crucial factors leading to cognitive impairment [20]. Silencing HCG27 inhibited the inflammatory response and oxidative stress in both MCAO rats and OGD/R-induced BV2 cells. The mNSS is used to determine neurological deficits [21], and silencing HCG27 could significantly reduce the mNSS score in MCAO rats. Cognitive dysfunction generally manifests as phenomena such as a decline in learning ability and memory [22]. Therefore, the MWM test revealed that the learning ability and memory of MCAO rats declined, whereas this phenomenon could be improved by silencing HCG27. Based on the above studies, we indicate that HCG27 could be involved in CI/R-induced cognitive dysfunction by promoting the inflammatory response and oxidative stress. In addition, while shRNA effectively knocked down HCG27, potential off-target effects cannot be fully excluded. Future studies using CRISPR-Cas9 knockout or antisense oligonucleotides will validate specificity.

lncRNAs participate in the pathogenesis of diverse diseases by regulating microRNAs (miRNAs) [23]. We have previously demonstrated that miR-27a-3p participates in the development of brain injury. It inhibited CI/R injury by directly targeting FOXO1 [24]. MiR-27a-3p participates in complications such as neuronal loss and neurological function impairment after intracerebral hemorrhage by targeting AQP11 [25]. Moreover, the overexpression of miR-27a-3p reversed the inflammatory response and neuronal apoptosis caused by the upregulation of the *Rgs1* gene [26]. XIST can be involved in CI/R injury through the miR-27a-3p/FOXO3 axis [27]. Consequently, we hypothesized that miR-27a-3p could be potentially implicated in CI/R-induced cognitive dysfunction. Thus, we confirmed the targeting relationship between HCG27 and miR-27a-3p through experiments. The level of miR-27a-3p diminished in MCAO rats, and increased with an increase in reoxygenation time in oxygen–glucose OGD/R-induced BV2 cells. In addition, the miR-27a-3p antagonist could reverse the inflammatory response, oxidative stress, cell injury, and apoptosis promoted by HCG27. HCG27 participated in CI/R injury-induced cognitive dysfunction by targeting miR-27a-3p.

The notable limitation is the exclusive use of BV2 microglial cells in vitro. While inflammation is a key component of CI/R injury, future studies should validate the HCG27/miR-27a-3p axis in primary neurons or neuron-glial co-culture models to address direct neuronal effects. In addition, given that miR-27a-3p has been shown to inhibit FOXO1-mediated oxidative stress, HCG27 may indirectly promote neuroinflammation by sequestering miR-27a-3p and upregulating FOXO1. This mechanistic link warrants exploration in future studies.

The current study primarily uses rodent models (MCAO rats) and mouse BV2 microglial cells, which may have species-specific limitations. In the future, we will employ human primary neural cells (such as cortical neurons and astrocytes) and induced pluripotent stem cell (iPSC)-derived microglia to validate the role of the HCG27/miR-27a-3p axis. This approach will clarify whether the regulatory mechanism of HCG27 in neuroinflammation and oxidative stress is conserved in humans, meeting the need for more clinically relevant cellular models. Furthermore, we will analyze serum and brain tissue samples from ischemic stroke patients to detect the expression levels of HCG27 and miR-27a-3p. By correlating these biomarkers with clinical parameters (such as stroke severity, cognitive function scores, and long-term prognosis), their translational significance can be established. Additionally, longitudinal studies tracking the dynamic changes of HCG27/miR-27a-3p after stroke may reveal their potential as diagnostic or prognostic indicators. To bridge the gap between rodent models and human diseases, we plan to use non-human primate models of cerebral ischemia-reperfusion injury. These models better recapitulate human cerebrovascular anatomy and pathophysiology, enabling the evaluation of HCG27-targeted therapies (such as shRNA-based gene silencing or miR-27a-3p mimics) in more clinically relevant contexts. This study shows that HCG27 affects neuroinflammation and oxidative stress by regulating miR-27a-3p, but its downstream signaling network remains undefined. Future research will explore whether HCG27/miR-27a-3p regulates known stroke-related pathways through transcriptomic and proteomic approaches, aiming to identify potential combinatorial therapeutic targets.

## Conclusions

Silencing HCG27 can alleviate the neurological damage caused by CI/R-induced cognitive dysfunction by promoting the function of miR-27a-3p, inhibiting apoptosis, inflammatory response, and oxidative stress. In addition, this study provided a new perspective for further research on cognitive dysfunction after cerebrovascular diseases.

## Electronic supplementary material

Below is the link to the electronic supplementary material.

Supplementary Material 1

## Data Availability

The datasets used and/or analysed during the current study are available from the corresponding author on reasonable request.
