# Supplementary material for: Silencing LncRNA HCG27 ameliorates cognitive dysfunction after ischemic stroke via miR-27a-3p regulation
Source: Hereditas. 2025 Jul 18;162:136. doi: 10.1186/s41065-025-00493-6 (PMC12275291; doi:10.1186/s41065-025-00493-6)
Supplement: Supplementary file 1 — Supplementary Material 1 [file 41065_2025_493_MOESM1_ESM.docx]

**Supporting Information Table S1** Primer sequences for Real-time PCR

| Gene | Primer sequence (5’-3’) |
| --- | --- |
| HCG27 | forward: CTTCCTTCCTGCCGCACCTTC |
|  | reverse: CAACCTCACCGAGAGCCCAAAC |
| miR-27a-3p | forward: GCGGGCGTTCACAGTGGCTA |
|  | reverse: CAGTGCAGGGTCCGAGGT |
| U6 | forward: CTCGCTTCGGCAGCACA |
|  | reverse: AACGCTTCACGAATTTGCGT |
| GAPDH | forward: TGTGGGCATCAATGGATTTGG |
|  | reverse: ACACCATGTATTCGGTCAAT |
